# John Howard Payne and Jenny Lind

**Published:** 1883-07

**Authors:** 


### JOHN HOWARD PAYNE AND JENNY LIND.

One who was present relates the following incident in the career of John Howard Payne : " Perhaps the most thrilling quarter of an hour of Payne's life was that when Jenny Lind sang 'Home, Sweet Home' to him. The occasion was the Jenny Lind concert in Washington the night of Dec. 17, 1850. The assemblage was, perhaps, the most distinguished ever seen in a concert room in the country. The immense National Hall, hastily constructed for the occasion on the ruins of the burned National Theatre, was filled to overflowing, notwithstanding the big price for admission, and the fact that the weather was cold and rainy. Among the notables present and occupying front seats were President Fillmore, Daniel Webster, Henry Clay, General Scott and John Howard Payne. Jenny Lind opened with the 'Casta Diva,' and followed with the 'Flute Song' (in which her voice contested rivalry for purity and sweetness with a flute in a duet), then the famous 'Bird Song,' and next on her programme the 'Greeting to America.' All the pieces were applauded apparently to the full capacity of an enthusiastic audience, and Mr. Webster, who was in his most genial after-dinner mood, emphasized the volume of plaudit by rising from his seat and making Jenny a profound bow, as if responding for the country to her 'Greeting.' But when the 'Swedish Nightingale' answered the encore by turning in the direction of John Howard Payne and giving 'Home, Sweet Home,' with all the wonderful tenderness, purity and simplicity fitting both the words and air of the immortal song, the difference was at once seen between the mechanical applause called out by a display of fine vocalization and that elicited by the 'touch of Nature that makes the whole world kin.' Before the first line of the song was completed the audience was fairly 'off its feet,' and could scarcely wait for a pause to give expression to its enthusiasm. People ordinarily of the undemonstrative sort clapped, stamped and shouted as if they were mad, and it seemed as if there would be no end to the uproar. Meantime all eyes were turned upon Payne, a small-sized, elegantly molded, gray-haired gentleman, who blushed violently at finding himself the centre of so many glances."

---

NEVER eat unless you are hungry.

IT is truth awaiting full recognition that the actual learning how to use the hands dexterously and accurately is a positive gain to the mental faculties. The trained eye and the trained hand are the best preparation for the trained thought. They give the first idea of system, order, accuracy, and the effective carrying out of a plan.

